# Outcome of patients aged 60‐75 years with newly diagnosed secondary acute myeloid leukemia: A single‐institution experience

**DOI:** 10.1002/cam4.2020

**Published:** 2019-06-07

**Authors:** Sarah Bertoli, Suzanne Tavitian, Pierre Bories, Isabelle Luquet, Eric Delabesse, Thibault Comont, Audrey Sarry, Françoise Huguet, Emilie Bérard, Christian Récher

**Affiliations:** ^1^ Service d'Hématologie Centre Hospitalier Universitaire de Toulouse Institut Universitaire du Cancer de Toulouse Oncopole Toulouse France; ^2^ Université Toulouse III Paul Sabatier Toulouse France; ^3^ Cancer Research Center of Toulouse UMR1037‐INSERM, ERL5294 CNRS Toulouse France; ^4^ Réseau Onco‐occitanie Institut Universitaire du Cancer de Toulouse Oncopole Toulouse France; ^5^ Laboratoire d'Hématologie Centre Hospitalier Universitaire de Toulouse Institut Universitaire du Cancer de Toulouse Oncopole Toulouse France; ^6^ Service de Médecine Interne Centre Hospitalier Universitaire de Toulouse Institut Universitaire du Cancer de Toulouse Oncopole Toulouse France; ^7^ Service d'Epidémiologie Centre Hospitalier Universitaire de Toulouse Toulouse France; ^8^ UMR 1027 INSERM‐Université de Toulouse III Toulouse France

**Keywords:** acute myeloid leukemia, azacitidine, CPX‐351, intensive chemotherapy, myelodysplasia‐related changes, secondary AML

## Abstract

A recent phase 3 trial showed that outcome of older patients with secondary acute myeloid leukemia (AML) may be improved by a liposomal encapsulation of cytarabine and daunorubicin (CPX‐351). This phase 3 study represents a unique example of prospective data in this rare subgroup providing basis for comparison with real life data. Here, we retrospectively assessed characteristics and outcome of patients aged 60‐75 years with secondary or therapy‐related AML in real life. Out of 218 patients that fulfilled CPX‐351 study criteria, 181 patients (83.0%) received antileukemic treatment either intensive chemotherapy (n = 121) or hypomethylating agents (HMA, n = 60). As compared with patients treated by chemotherapy, HMA‐treated patients were older, had lower WBC, more often AML with antecedent myelodysplastic syndrome and adverse cytogenetic risk. In chemotherapy‐treated patients, the complete response rate was 69%, median overall survival (OS) was 11 months whereas 3‐year and 5‐year OS was 21% and 17%, respectively. In HMA‐treated patients, the complete response rate was 15%, median OS was 11 months whereas 3‐year and 5‐year OS was 15% and 2%, respectively. In conclusion, although outcome of older patients with high‐risk AML is very poor, a significant proportion of patients treated by standard intensive chemotherapy but not HMA are long‐term survivors.

## 1 INTRODUCTION

The majority of acute myeloid leukemia (AML) arise de novo.^1^ However, in approximately 25% of cases, AML are diagnosed in patients with antecedent of hematological disorders or cytotoxic therapies. This subgroup of so‐called secondary AML is heterogeneous, including therapy‐related AML (t‐AML) which occur after prior exposure to cytotoxic chemotherapy and/or radiotherapy and secondary AML (sAML) which occur in the course of a previous myeloid disease such as myelodysplastic syndrome (MDS), chronic myelo‐monocytic leukemia (CMML) or Philadelphia‐negative myeloproliferative neoplasia (MPN).^2^ Both t‐AML and sAML do have a very poor prognosis compared to de novo AML and probably, one of the poorest prognosis in oncology especially in patients >60 years.^3^, ^4^, ^5^ Many well‐known adverse factors more frequently observed in such patients can explain this outcome including older age, comorbidities, multidrug resistance phenotype, adverse cytogenetics and molecular abnormalities.^6^ Until recently, therapeutic strategies did not really differ from de novo AML. Patients are offered induction chemotherapy and allogeneic stem‐cell transplantation if deemed fit for intensive therapies, those considered unfit for such treatment receive hypomethylating agents (HMA), low dose cytarabine or supportive care.^3^, ^4^, ^7^

The CPX‐351 trial has been specifically designed for older patients with secondary or high‐risk AML and demonstrated the superiority of the dual‐drug liposomal encapsulation of daunorubicin and cytarabine (CPX‐351) over the conventional “7 + 3” cytarabine‐daunorubicin regimen.^8^ This phase 3 study represents a unique example of prospective data in this rare AML subgroup providing a solid basis for comparison with real life data. Therefore, the objective of this study was to describe the outcome of older AML patients fulfilling main criteria of the CPX‐351 trial in order to assess the impact of current standard treatments used in routine at our institution in this specific patient population.

## 2 SUBJECTS AND METHODS

### 2.1 Patient's selection

Selection criteria for this retrospective study were a newly diagnosis of AML according to WHO criteria^2^ (excluding acute promyelocytic leukemia and core binding factor AML) between January 1st, 2000 and December 31st 2016 included in the AML database of Toulouse University Hospital/IUCT‐Oncopole and: 60‐75 years of age, Eastern Cooperative Oncology Group (ECOG) performance status 0‐2, no previous treatment for AML with the exception of hydroxyurea, serum creatinine <176 μmol L^−1^, serum total bilirubin <34 μmol L^−1^ prior history of MDS (post‐MDS AML), CMML (post‐CMML AML) or exposure to cytotoxic therapy or ionizing radiotherapy for an unrelated disease (t‐AML), or de novo AML with MDS‐related cytogenetic abnormalities or AML with multilineage dysplasia (and no NPM1 or CEBPA mutation). Post‐MPN AML and patients with clinical evidence of active central nervous system leukemia were excluded.^8^ Written informed consent was obtained in accordance with the Declaration of Helsinki, allowing the collection of clinical data in the anonymized database. Cytogenetic risk classification was defined according to the MRC classification.^9^ Comorbidities were collected according to Charlson comorbidity index definition.^10^ Regimen of intensive induction chemotherapy and azacitidine have been previously described.^3^, ^11^

### 2.2 Response criteria and endpoints

Bone marrow (BM) assessment in patients treated by intensive chemotherapy was performed after blood recovery or in case of delayed recovery, between days 35 and 45. In the azacitidine group, BM aspiration was carried out after 3‐6 cycles. Response to treatment, early death (ED), relapse‐free survival (RFS), event‐free survival (EFS), and overall survival (OS) were defined according to the EuropeanLeukemia Net (ELN) 2017 criteria.^12^

### 2.3 Statistical analyses

Statistical analyses were performed using STATA software 14.2 (STATA Corp., College Station, TX, USA). We first described characteristics of patients using the appropriate descriptive statistics according to the type of variables. Descriptive statistics included median with interquartile range (IQR) for continuous variables and number of nonmissing observations with frequency (%) for categorical variables. We then compared the characteristics of the patients treated with intensive chemotherapy vs hypomethylating agents. Categorical variables were compared between groups using the *χ* ^2^‐test (or Fisher's exact test when necessary). Student's *t* test was used to compare the distribution of continuous data (Mann‐Whitney's test was used when the distribution departed significantly from normality or when homoscedasticity was rejected). OS was described in patients treated with intensive chemotherapy vs hypomethylating agents using Kaplan‐Meier survival curves. Prognostic factors independently associated with OS in patients treated with intensive chemotherapy vs hypomethylating agents were assessed using Cox modeling. Age, ECOG performance status, Charlson comorbidity index, AML subtype, antecedent of chemotherapy, white cell count, albumin, serum ferritin, bone marrow blasts, and cytogenetic risk at diagnosis together with type of chemotherapy and allogeneic stem‐cell transplantation (only for patients treated with intensive chemotherapy) were assessed as potential prognostic factors. Variables initially introduced into the multivariate survival analyses were all variables associated with OS in univariate analyses with a *P*‐value <0.20. A backward analysis was then applied until only variables significantly and independently associated with OS (*P*‐value <0.05) remained. The proportional‐hazard assumption was tested for each covariate of the Cox model using the “log‐log” plot method curves and was always met. When the linearity hypothesis was not respected, continuous variables were transformed into ordered data. Interactions between independent covariates were tested in the final models. None were significant. Allogeneic stem‐cell transplantation was evaluated as a time‐dependent covariate. All reported *P*‐values were two‐sided and the significance threshold was set at <0.05.

## 3 RESULTS

### 3.1 Study population

Out of 2090 newly diagnosed AML patients included in our database between 2000 and 2016, 748 were aged 60‐75 years and 218 patients fulfilled the inclusion criteria for this study. Their characteristics are presented in Table 1. Molecular data are shown in Table S1. The male/female sex ratio was 1.6. There were 51 t‐AML (23.4%), 60 post‐MDS AML (27.5%), 13 post‐CMML AML (5.9%), 78 de novo AML with MDS‐like karyotype (35.8%) and 16 AML with multilineage dysplasia (7.3%). Twenty patients were previously exposed to HMA treatment. One hundred and eighty‐one patients (83.0%) were selected to receive an anti‐AML treatment either intensive chemotherapy (n = 121) or HMA (n = 60), mainly azacitidine (n = 57). As expected, HMA‐treated patients were older, had lower WBC, more often AML with antecedent MDS and adverse cytogenetic risk compared with patients treated by intensive chemotherapy.^3^, ^13^

**Table 1:** Characteristics of the 218 older (60‐75 years) AML patients with high‐risk AML

Characteristics	Study population^a^ N = 218	Intensive chemotherapy N = 121	Hypomethylating agents N = 60	*P*‐value^b^
Sex – n. (%)				
Male	134 (61.5)	72 (59.5)	37 (61.7)	0.780
Female	84 (38.5)	49 (40.5)	23 (38.3)	
Age – years				
Median (IQR)	68.9 (64.9‐72.6)	67.2 (63.2‐71.0)	71.2 (67.2‐74.3)	<0.001
60‐69 – no. (%)	123 (56.4)	84 (69.4)	26 (43.3)	
70‐75 – no. (%)	95 (43.6)	37 (30.6)	34 (56.7)	
ECOG performance status – n. (%)				
0	51 (23.4)	35 (28.9)	13 (21.7)	0.089
1	125 (57.3)	71 (58.7)	32 (53.3)	
2	42 (19.3)	15 (12.4)	15 (25.0)	
Charlson comorbidity index ‐ n. (%)				
0	125 (59.0)	71 (61.7)	34 (56.7)	0.516
≥1	87 (41.0)	44 (38.3)	26 (43.3)	
Extramedullary involvement – n. (%)				
No	172 (80.0)	88 (73.9)	55 (91.7)	0.005
Yes	43 (20.0)	31 (26.1)	5 (8.3)	
AML subtype – n. (%)				
Therapy‐related AML	51 (23.4)	29 (24.0)	15 (25.0)	0.067
Prior chemotherapy alone	12 (5.5)	5 (4.1)	4 (6.7)	
Prior chemotherapy and radiotherapy	24 (11.0)	15 (12.4)	5 (8.3)	
Prior radiotherapy alone	15 (6.9)	9 (7.4)	6 (10.0)	
AML with antecedent MDS	60 (27.5)	22 (18.2)	21 (35.0)	
With prior HMA	20 (9.2)	1 (0.8)	7 (11.7)	
Without prior HMA	36 (16.5)	21 (17.3)	14 (23.3)	
AML with antecedent CMML	13 (6.0)	9 (7.4)	1 (1.7)	
De novo AML with MDS karyotype	78 (35.8)	48 (39.7)	20 (33.3)	
Multilineage dysplasia	16 (7.3)	13 (10.7)	3 (5.0)	
Patients with prior HMA exposure – n. (%)	20 (9.2)	1 (0.8)	7 (11.7)	0.003
Patients with antecedent of chemotherapy– n. (%)	65 (29.8)	24 (19.8)	16 (26.7)	0.297
Infection at diagnosis – n. (%)				
No	167 (77.7)	92 (77.3)	49 (81.7)	0.501
Yes	48 (22.3)	27 (22.7)	11 (18.3)	
White cell count – giga/liter				
Median (IQR)	4.3 (1.7‐18.1)	6.6 (2.5‐25.5)	2.4 (1.3‐9.4)	<0.001
Platelet count – giga/liter				
Median (IQR)	53.0 (28.0‐100.0)	56.0 (35.0‐93.0)	76.5 (30.5‐114.0)	0.436
Bone marrow blasts – %				
Median (IQR)	40.5 (28.0‐65.0)	48.0 (33.0‐75.0)	32.0 (23.0‐44.0)	<0.001
Cytogenetic risk – n. (%)				
Intermediate	105 (48.4)	68 (56.2)	24 (40.0)	0.040
Adverse	112 (51.6)	53 (43.8)	36 (60.0)	
Albumin ‐ g/liter				
Median (IQR)	38.0 (35.0‐42.0)	38.0 (35.0‐42.0)	39.0 (36.0‐42.0)	0.578
Normal – n. (%)	156 (75.4)	94 (79.7)	46 (80.7)	0.872
Low – n. (%)	51 (24.6)	24 (20.3)	11 (19.3)	
LDH – UI/liter				
Median (IQR)	560.0 (364.0‐897.0)	615.0 (411.0‐1136.0)	464.5 (316.0‐680.0)	0.001
Normal – n. (%)	61 (28.4)	29 (24.0)	21 (36.2)	0.088
>Normal– n. (%)	154 (71.6)	92 (76.0)	37 (63.8)	
Creatinine ‐ μmol L^−1^				
Median (IQR)	86.0 (70.0‐101.0)	86.0 (72.0‐99.0)	84.5 (74.0‐104.5)	0.759
Bilirubin ‐ μmol L^−1^				
Median (IQR)	9.9 (7.0‐14.0)	10.1 (7.0‐13.8)	9.0 (6.7‐12.5)	0.116
Serum ferritin ‐ μg/liter				
Median (IQR)	839.0 (421.0‐1636.0)	679.0 (398.0‐1287.0)	725.0 (323.0‐1709.0)	0.741
Fibrinogen – g/liter				
Median (IQR)	4.1 (3.2‐4.8)	4.1 (3.1‐4.8)	4.1 (3.4‐4.7)	0.504
Chemotherapy – n. (%)				
Ida‐AraC		21 (17.4)		
Ida‐AraC‐lomustine		92 (76.0)		
Ida‐AraC‐GO		5 (4.1)		
Other^c^		2 (1.7)		
Allo‐SCT – n. (%)				
No		109 (90.1)	58 (96.7)	0.147
Yes		12 (9.9)	2 (3.3)	

ECOG, Eastern Cooperative Oncology Group; HMA, hypomethylating agents; MDS, myelodysplastic syndrome; CMML, chronic myelomonocytic leukemia; Ida, idarubicin; GO, gemtuzumab ozogamycin.

a Patients (n = 37) who did not receive intensive chemotherapy or hypomethylating agents (HMA) were treated by supportive care (n = 22), low dose cytarabine (n = 10) or others (n = 5). Among the 22 BSC patients (median age, 68 years; median OS, 71 days), 11 had post‐MDS AML previously treated by HMA for higher‐risk MDS, 5 had complex/monosomal karyotype, 3 had t‐AML (2 with complex karyotype), 1 had post‐CMML AML with complex caryotype and 1 post‐MDS AML.

b Intensive chemotherapy vs hypomethylating agents.

c Time Sequential induction (n = 1), FLAG (fludarabin‐AraC‐GCSF) (n = 1).

### 3.2 Outcome of patients treated by intensive chemotherapy

**Figure 1:**
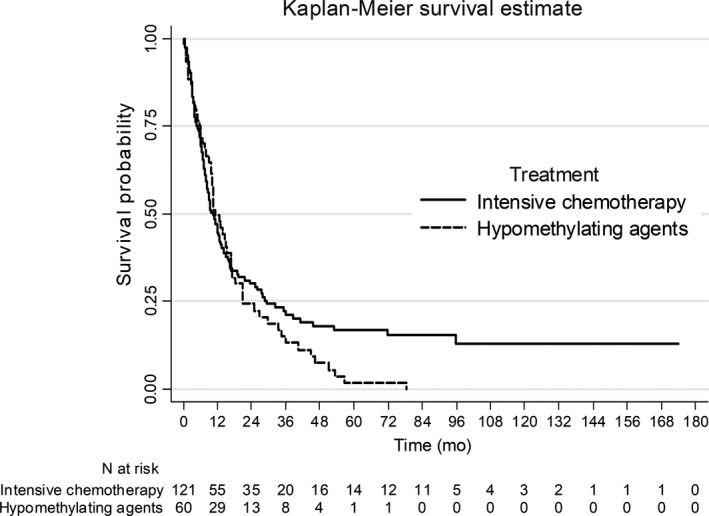
Overall survival of patients treated by intensive chemotherapy or hypomethylating agents

Among the 121 patients treated by intensive chemotherapy, early death occurred in 5 (4.1%) and 12 (9.9%) patients at day 30 and day 60, respectively. CR/CRi was achieved in 84 patients (69.4%). Main adverse events occurred during induction therapy are described in Table 2. As post‐remission treatment, 12 patients (9.9%) underwent allogeneic‐stem cell transplantation. With a median follow‐up of nondeceased patients of 67 months (IQR, 26‐95), 54 patients in first CR/CRi relapsed (64.3%). Median EFS, RFS and OS were 7 (IQR, 3‐16), 8 (4–28), and 11 (5‐29) months, respectively (Figure 1). OS was 21% [95%CI: 14‐29] and 17% [10‐25] at 3 and 5 years, respectively. In allografted patients, median OS starting from allogeneic stem cell transplantation was 15 months (5‐NR) whereas 3‐year and 5‐year OS were 50% [95%CI: 21‐74]. Multivariate analysis showed that antecedent of chemotherapy (HR, 2.41; 95%CI: 1.48‐3.92; *P *<* *0.001), adverse cytogenetic risk (HR, 1.90; 95%CI: 1.26‐2.88; *P *=* *0.002) and allogeneic stem cell transplantation evaluated as a time‐dependent variable (HR, 0.38; 95%CI: 0.16‐0.89; *P *=* *0.027) were significantly and independently associated with overall survival.

**Table 2:** Response and adverse events after intensive chemotherapy or hypomethylating agents

	Intensive chemotherapy^a^ N = 121	Hypomethylating agents^b^ N = 60
Overall response (CR+CRi) – n (%)		
No	37 (30.6)	51 (85.0)
Yes	84 (69.4)	9 (15.0)
Deaths at day 30 – n (%)		
No	116 (95.9)	57 (95.0)
Yes	5 (4.1)	3 (5.0)
Deaths at day 60 – n (%)		
No	109 (90.1)	52 (86.7)
Yes	12 (9.9)	8 (13.3)
Bacterial infections ‐ n (%)		
No	79 (65.3)	27 (45.0)
Yes	37 (30.6)	30 (50.0)
Fungal infections ‐ n (%)		
No	94 (77.7)	53 (88.3)
Yes	22 (18.2)	4 (6.7)
Bleeding events (grade 3‐4) ‐ n (%)		
No	113 (93.4)	54 (90.0)
Yes	3 (2.5)	3 (5.0)

CR, complete response; CRi, complete response with incomplete blood recovery.

a Adverse events during induction chemotherapy.

b Adverse events during HMA treatment (all courses).

### 3.3 Outcome of patients treated by HMA

Among the 60 patients treated with HMA, early death occurred in 3 (5.0%) and 8 (13.3%) patients at day 30 and day 60, respectively. Patients received a median number of 6.5 cycles (4.0‐14.5) of HMA. CR/CRi was achieved in 9 patients (15%). Main adverse events occurred over the treatment period are described in Table 2. Only 2 patients underwent allogeneic stem cell transplantation. With a median follow‐up of non‐deceased patients of 13 months (IQR, 6‐20), 5 patients in first CR/CRi relapsed (55.6%). Median OS was 11 (6‐21) months (Figure 1B). OS was 15% [95%CI: 7‐26] and 2% [0.2‐8.7] at 3 and 5 years, respectively. Multivariate analysis showed that a normal albumin value (HR, 0.38; 95%CI: 0.18‐0.79; *P *=* *0.010) and bone marrow blasts >30% (HR, 1.85; 95%CI: 1.06‐3.24; *P *=* *0.032) were significantly and independently associated with overall survival.

### 3.4 Subgroup analysis

After censoring patients at the date of allogeneic stem cell transplantation, OS at 5 years was 14% [8‐22] and 2% [0.2‐9.3], in patients treated by intensive chemotherapy and HMA, respectively. In patients aged ≥ 70 years at diagnosis, OS at 5 years was 11% [3‐25] and 3% [0.3‐14.3], in patients treated by intensive chemotherapy and HMA, respectively; whereas in patients aged < 70 years, OS at 5 years was 19% [11‐29] and 0%, in patients treated by intensive chemotherapy and HMA, respectively.

After excluding AML with multilineage dysplasia form the analyses, among the 108 patients treated by intensive chemotherapy, early death occurred in 5 (4.6%) and 12 (11.1%) patients at day 30 and day 60, respectively. CR/CRi was achieved in 75 patients (69.4%). Median EFS, RFS and OS were 7 (IQR, 3‐16), 8 (4‐31) and 9.5 (4‐29) months, respectively. OS was 20% [95%CI: 12‐28] and 17% [10‐25] at 3 and 5 years, respectively. Among the 57 patients treated with HMA, early death occurred in 3 (5.3%) and 8 (14.0%) patients at day 30 and day 60, respectively. CR/CRi was achieved in 9 patients (15.8%). Median OS was 11 (6‐21) months. OS was 16% [95%CI: 8‐27] and 2% [0.2‐9.3] at 3 and 5 years, respectively.

Lastly, 15 patients were 5‐year survivors (n = 14, intensive chemotherapy and N = 1, HMA). Among them, 4 (27%) patients received allogeneic stem cell transplantation, 12 (80%) patients were aged < 70 years at diagnosis and 9 (60%) vs 6 (40%) had an intermediate or adverse cytogenetic risk. The characteristics of these long‐term survivors are shown in Table 3.

**Table 3:** Main characteristics of the 15 5‐year survivors

Characteristics	5‐year survivors N = 15
Sex – n. (%)	
Male	7 (46.7)
Female	8 (53.3)
Age – years	
Median (IQR)	65.5 (61.9‐69.0)
60‐69 – no. (%)	12 (80.0)
70‐75 – no. (%)	3 (20.0)
ECOG performance status – n. (%)	
0	2 (13.3)
1	11 (73.3)
2	2 (13.3)
Charlson comorbidity index ‐ n. (%)	
0	11 (73.3)
≥1	4 (26.7)
Extramedullary involvement – n. (%)	
No	12 (80.0)
Yes	3 (20.0)
AML subtype – n. (%)	
Therapy‐related AML	4 (26.7)
AML with antecedent MDS	2 (13.3)
AML with antecedent CMML	0 (0.0)
De novo AML with MDS karyotype	8 (53.3)
Multilineage dysplasia	1 (6.7)
White cell count – giga/liter	
Median (IQR)	16.4 (2.5‐29.8)
Platelet count – giga/liter	
Median (IQR)	78.0 (41.0‐178.0)
Bone marrow blasts – %	
Median (IQR)	75.0 (48.0‐86.0)
Cytogenetic risk – n. (%)	
Intermediate	9 (60.0)
Adverse	6 (40.0)
Albumin ‐ g/liter	
Median (IQR)	38.0 (37.0‐41.0)
Normal – n. (%)	14 (93.3)
Low – n. (%)	1 (6.7)
LDH – UI/liter	
Median (IQR)	831.0 (520.0‐1206.0)
Normal – n. (%)	1 (6.7)
>Normal– n. (%)	14 (93.3)
Creatinine ‐ μmol L^−1^	
Median (IQR)	84.0 (64.0‐104.0)
Bilirubin ‐ μmol L^−1^	
Median (IQR)	9.8 (7.0‐15.7)
Treatment – n. (%)	
Chemotherapy	14 (93.3)
HMA	1 (6.7)
Allo‐SCT – n. (%)	
Post‐chemotherapy	4 (28.6)
Post‐HMA	0 (100.0)

## 4 DISCUSSION

This study shows that among older patients with secondary or high‐risk AML, intensive chemotherapy may achieve a fairly good response rate and a median OS of 11 months. These results compare favorably with the control chemotherapy arm of the CPX‐351 trial which consisted of three daily doses of daunorubicin 60 mg/m² and seven daily doses of cytarabine 100 mg/m².^8^ This schema is considered as the standard of care according to current guidelines.^12^ In this trial, this “3 + 7” regimen induced an overall response rate (CR/CRi) of 33.3% with early mortality rates of 10.6% and 21.2% at day 30 and day 60, respectively. Median EFS and OS were 1.31 and 5.95 months, respectively.

Our study dealing with high‐risk patients selected in a real life database suggests that better results have been achieved with our chemotherapy regimen. However, although we have used the phase 3 criteria to select our study population, there are some differences in the characteristics of patients from both studies that could explain the poorest results of the standard chemotherapy used in the phase 3 trial. Probably, the most important difference was the higher number of patients of the phase 3 that were previously exposed to HMA (45.5% vs 0.8%). Those patients who progressed from MDS to AML during HMA treatment have a particularly poor outcome.^14^ In our study population, the proportion of patients who had been exposed to HMA was extremely low compared to the CPX‐351 study. In Europe, azacitidine is used only in patients with higher‐risk MDS. Those patients are poor candidates to intensive chemotherapy especially when they have been exposed to several cycles of azacitidine before progressing to AML. In this situation, we favor clinical trials with new agents when available, supportive care or allogeneic stem cell transplantation for the few patients that are fit enough to receive such treatment. There were also more de novo AML with MDS karyotype in our study population. This subgroup includes very rare chromosomal abnormalities whose prognostic value is likely heterogeneous and not always associated with chemoresistance.^2^ Moreover, it does not include other high‐risk chromosomal abnormalities including 11q23 rearrangements, inv3/t(3;3)(q21.3;q26.2), or t(6;9)(p23;q34.1) that belong to AML with recurrent genetic abnormalities, another distinct subgroup of the WHO 2016 classification. Lastly, the proportion of patients with intermediate cytogenetic risk who received intensive chemotherapy in our real life study was higher than in the control arm of the phase 3 CPX‐351 trial (56.2% vs 39.7%). As confirmed in our multivariate analysis, cytogenetic risk remains a strong prognostic factor in secondary AML treated by intensive chemotherapy.

As far as intensive chemotherapy is concerned, our standard of care for older AML patients combines lomustine, idarubicin, and cytarabine.^15^, ^16^ Lomustine is an alkylating agent with anti‐leukemic activity.^17^ The FILO study group recently reported the significant impact of adding lomustine to idarubicin and cytarabine with a higher response rate and reduction in relapses resulting in better EFS and improved OS.^18^ Furthermore, we have been using idarubicin over 5 days for a long time in both younger and older patients based on the pharmacologic properties of this drug.^19^ Notably, we have previously reported a randomized phase 3 trial showing that idarubicin used according this schema (8 mg/m²/d for 5 days) improved EFS, DFS and OS in younger patients with AML compared to daunorubicin used at a daily dose of 60 mg/m² for 3 days.^20^ Idarubicin is a 4‐demethoxy‐anthracycline analogue of daunorubicin with increased lipophilia and better cellular uptake compared to daunorubicin. Idarubicin also displays a lower susceptibility to multi‐drug resistance and a stronger binding to DNA resulting in a 10‐fold higher cytotoxic activity when compared to daunorubicin. Moreover, its primary metabolite, idarubicinol which demonstrates similar activity to idarubicin in vitro, is still detectable in plasma at least 72 hours following intravenous infusion of idarubicin by contrast to daunorubicin's lower half‐life.^21^, ^22^, ^23^ Beside the dose effect, the duration of exposition to idarubicin given over a 5‐day period could also induce a deeper antileukemic effect than daunorubicin given over 3 days. In some way, this is reminiscent of the mechanism of action of CPX‐351 who significantly prolongs exposure to anthracyclines.^24^, ^25^ It is thus this tempting to speculate that, in this high‐risk population, adding lomustine and using idarubicin in a prolonged manner could provide higher antileukemic activity as compared to the classical 3 + 7 used in the CPX‐315 phase 3 trial. Moreover, multivariate analysis in chemotherapy treated patients also highlighted the role of allogeneic stem cell transplantation that should remain the standard of care in this high‐risk population.

In this study, we have observed a high rate of invasive fungal infections (18.2%) in chemotherapy‐treated patients despite prophylaxis with voriconazole or posaconazole which can be related to the selection of high‐risk AML. Indeed, we have previously reported that 50% of AML patients with invasive aspergillosis had an adverse prognosis according to their cytogenetic features and most patients who died of invasive aspergillosis‐related complications were refractory to chemotherapy. Furthermore, patients with low‐risk cytogenetics did not (or anecdotally) develop invasive aspergillosis, suggesting that rapid and more effective disease control of leukemia is a determining factor in the incidence and outcome of invasive fungal infections.^26^

HMA have become very popular in the management of older patients with high‐risk or secondary AML providing responses, clinical benefit, and better quality of life.^27^, ^28^ In a post‐hoc analysis of the AZA‐AML 001 trial, median OS among all azacitidine‐treated patients with AML with myelodysplasia‐related changes was 8.9 months whereas the 52 patients aged 65‐74 years had a median OS of 14.2 months.^29^ This is similar to the 60 patients treated in our real life cohort. This median OS compares favorably with intensive chemotherapy. However, although HMA‐treated patients were older and had more often AML with antecedent myelodysplastic syndrome or adverse cytogenetic risk, it should be noted that there were few patients alive at 3 years (15%) and virtually none at 5 years (2%) as compared to patients treated by intensive chemotherapy (21% and 17% at 3 and 5 years, respectively). In the CPX‐351 trial, the 2‐years OS was 12.3% and 31.1% in high‐risk patients treated by “3 + 7” and CPX‐351, respectively.^8^ The median follow‐up of this study is too short to provide long‐term outcome of those patients.

Obviously, our study has several limitations to make definitive general conclusions. It was retrospective, monocentric and we recognize that our chemotherapy regimen is not broadly used outside centers from the French Innovative Leukemia Organization (FILO) study group.^18^ Moreover, despite the selection criteria of CPX‐351 trial, there were significant differences between both studies especially regarding prior exposure to HMA, which should be kept in mind for interpretation of data.

Our study focused on a patient population susceptible to be selected in real life to receive CPX‐351. The use of CPX‐351 in older patients with high‐risk AML is appealing with respect to safety and efficacy including patients who benefit from allogeneic stem cell transplantation. Although we cannot strictly compare the results of the CPX‐351 trial nor those of the AZA‐AML 01 trials with our real life cohort, our results suggest that a randomized clinical trial assessing safety and long‐term outcome comparing CPX‐351, a more intensive chemotherapy schema than “3 + 7” and eventually, azacitidine would be interesting to help clinicians in the therapeutic choice for this very hard‐to treat population.

## CONFLICT OF INTEREST

C. Récher has received research grants from Amgen, Novartis, Celgene, Jazz Pharma and Sunesis and is an advisor for Abbvie, Sunesis, Janssen, Jazz, Novartis, Celgene, Macrogenics and Pfizer. F. Huguet is an advisor for Amgen, BMS, Cellgene, Incyte, Jazz Pharma, Novartis, Pfizer. S. Tavitian and E. Delabesse are advisors for Novartis. P. Bories is an advisor for Sanofi and Novartis. S. Bertoli is an advisor for Sanofi and Astellas.
