# Supplementary material for: Outcome of patients aged 60‐75 years with newly diagnosed secondary acute myeloid leukemia: A single‐institution experience
Source: Cancer Med. 2019 Jun 7;8(8):3846–54. doi: 10.1002/cam4.2020 (PMC6639188; doi:10.1002/cam4.2020)
Supplement: Supplementary file 1 [file CAM4-8-3846-s001.docx]

**Supplementary Table 1 : molecular characteristics.**

| **Characteristics** | **Study population**  **N= 218** | **Intensive chemotherapy**  **N= 121** | **Hypomethylating agents**  **N= 60** |
| --- | --- | --- | --- |
| **FLT3-ITD – n. (%)**  Yes  No | N=126  13 (10.3)  113 (89.7) | 11 (12.2)  79 (87.9) | 1 (5.0)  19 (95.0) |
| **FLT3-TKD – n. (%)**  Yes  No | N=33  2 (6.1)  31 (93.9) | 2 (7.1)  26 (92.9) | 0 (0.0)  1 (100.0) |
| **NPM1 – n. (%)**  Yes  No | N=115  14 (12.2)  101 (87.8) | 13 (16.3)  67 (83.8) | 1 (5.0)  19 (95.0) |
| **CEBPA – n. (%)**  Yes  No | N=38  1 (2.6)  37 (97.4) | 1 (3.1)  31 (96.9) | 0 (0.0)  2 (100.0) |
| **IDH1^R132^**  Yes  No | N=65  1 (1.5)  64 (98.5) | 1 (2.1)  47 (97.9) | 0 (0.0)  11 (100.0) |
| **IDH2^R140^**  Yes  No | N=65  6 (9.2)  59 (90.8) | 5 (10.4)  43 (89.6) | 1 (9.1)  10 (90.9) |
| **IDH2^R172^**  Yes  No | N=65  2 (3.1)  63 (96.9) | 2 (4.2)  46 (95.8) | 0 (0.0)  11 (100.0) |
| **DNMT3A**  Yes  No | N=25  2 (8.0)  23 (92.0) | 2 (10.5)  17 (89.5) | 0 (0.0)  6 (100.0) |
| **TET2**  Yes  No | N=22  4 (18.2)  21 (87.5) | 4 (19.0)  17 (81.0) | 0 (0.0)  1 (100.0) |
| **NRAS**  Yes  No | N=24  3 (12.5)  20 (90.9) | 2 (11.1)  16 (88.9) | 1 (20.0)  4 (80.0) |
| **KRAS**  Yes  No | N=22  2 (9.1)  20 (90.9) | 2 (10.5)  17 (89.5) | 0 (0.0)  2 (100.0) |
